# Trait and State Anxiety Effects on Mismatch Negativity and Sensory Gating Event-Related Potentials

**DOI:** 10.3390/brainsci13101421

**Published:** 2023-10-07

**Authors:** Vasileios Ioakeimidis, Laura Lennuyeux-Comnene, Nareg Khachatoorian, Sebastian B. Gaigg, Corinna Haenschel, Marinos Kyriakopoulos, Danai Dima

**Affiliations:** 1 Department of Psychology, School of Health and Psychological Sciences, City University of London, 10 Northampton Square, London EC1V 0HB, UK; ioakeimidisv@cardiff.ac.uk (V.I.); laura.lennuyeux-comnene.2@city.ac.uk (L.L.-C.); s.b.gaigg@city.ac.uk (S.B.G.); corinna.haenschel.1@city.ac.uk (C.H.); 2 South London and the Maudsley NHS Foundation Trust, London SE5 8AF, UK; 3 Department of Child and Adolescent Psychiatry, Institute of Psychiatry, Psychology and Neuroscience, King’s College London, London SE5 8AF, UK; 4 1st Department of Psychiatry, National and Kapodistrian University of Athens, 11528 Athens, Greece; 5 Department of Neuroimaging, Institute of Psychiatry, Psychology and Neuroscience, King’s College London, London SE5 8AF, UK

**Keywords:** attention, salience, predictive coding, change detection, inhibition

## Abstract

We used the auditory roving oddball to investigate whether individual differences in self-reported anxiety influence event-related potential (ERP) activity related to sensory gating and mismatch negativity (MMN). The state-trait anxiety inventory (STAI) was used to assess the effects of anxiety on the ERPs for auditory change detection and information filtering in a sample of thirty-six healthy participants. The roving oddball paradigm involves presentation of stimulus trains of auditory tones with certain frequencies followed by trains of tones with different frequencies. Enhanced negative mid-latency response (130–230 ms post-stimulus) was marked at the deviant (first tone) and the standard (six or more repetitions) tone at Fz, indicating successful mismatch negativity (MMN). In turn, the first and second tone in a stimulus train were subject to sensory gating at the Cz electrode site as a response to the second stimulus was suppressed at an earlier latency (40–80 ms). We used partial correlations and analyses of covariance to investigate the influence of state and trait anxiety on these two processes. Higher trait anxiety exhibited enhanced MMN amplitude (more negative) (*F*_(1,33)_ = 14.259, *p* = 6.323 × 10^−6^, *η_p_^2^* = 0.302), whereas state anxiety reduced sensory gating (*F*_(1,30)_ = 13.117, *p* = 0.001, *η_p_^2^* = 0.304). Our findings suggest that high trait-anxious participants demonstrate hypervigilant change detection to deviant tones that appear more salient, whereas increased state anxiety associates with failure to filter out irrelevant stimuli.

## 1. Introduction

Mismatch negativity (MMN) is an event-related potential (ERP) that is generated by the presentation of an unusual (deviant) stimulus preceded by a series of regular (standard) ones in the electroencephalogram (EEG). It occurs at approximately 200 ms post-stimulus, with frontocentral topography on the midline of the scalp [1]. In the auditory modality, the MMN wave is said to reflect a pre-attentive mechanism of sensory memory trace formation [2]. It is generated as a result of the brain’s failure to predict the auditory input, based on its mismatch with the encoded memory trace [2]. In the context of the predictive coding framework, a repeated tone becomes standard and is predicted by top-down prior expectations [3]. This standard tone elicits a heightened ERP amplitude in the EEG signal around 130–230 ms. At the presence of an oddball stimulus, the mismatch between the prior expectations and the actual sensory input results in a downward—more negative—ERP wave. The MMN is the difference wave between the deviant and the standard tone, demonstrating a negative wave. This is said to reflect a mechanism of change detection in the acoustic environment. In this paper, we are utilizing the auditory roving oddball paradigm, in which the physical properties of the auditory stimuli cannot influence the MMN response, as the deviants and standards have identical characteristics and stimulus frequency [4,5].

Individuals with high trait anxiety demonstrate larger (more negative) MMN responses [6,7] in the EEG. Similarly, individuals with high anticipatory anxiety [8], as well as individuals with anxiety disorders, such as post-traumatic stress disorder (PTSD), display enhanced MMN [9,10,11] and panic disorder [12]. Increased MMN in the oddball task in individuals with heightened anxiety suggests that it is specific to the neural mechanisms sensitive to stimulus change. Detecting unusual, salient, and possibly dangerous changes in one’s surroundings is a fundamental ability that helps ensure the survival of different species. Enhanced MMN as a function of anxiety supports the hypothesis that environmental irregularities may play a significant role in the cognitive and neurophysiological characteristics that are observed in preclinical self-reported anxiety [13,14,15]. Hypervigilance, hyperarousal, inability to stop or control worrying, and feelings of nervousness are commonly reported by those who score high in anxiety self-report instruments. It is possible that the root of these experiences may be found, to some degree, in pre-attentive abnormalities manifested in the MMN generation.

Sensory gating is an earlier pre-attentive process that occurs as early as 50–100 ms post-stimulus presentation and enables the brain to suppress redundant sensory information, such as repetitive auditory stimuli. It can be tested with the presentation of identical tones as in the case of the paired click [16] or the oddball paradigm, utilized in this study [5,17]. The brain response usually exhibits a positive potential to the first new tone (S1), whereas it is inhibited in subsequent repetition of an identical tone (S2), suggested to act as a protective mechanism against sensory flooding of higher association areas [17]. While both sensory gating and MMN are involved in the brain’s response to auditory stimuli, they address different aspects of sensory processing. Sensory gating focuses on the attenuation of redundant stimuli to prevent information overload, whereas MMN is concerned with the brain’s ability to detect and process unexpected changes in the sensory environment.

Individuals with anxiety disorders exhibit impairments in auditory sensory gating expressed as failure to gate out the auditory stimuli, by exhibiting larger S2 amplitudes, for example, in individuals with panic disorder [18,19], PTSD [20], and obsessive compulsive disorder [21]. S2 amplitude-driven differences in sensory gating indicate lack of inhibition to the repeating stimulus [22]. Additionally, sensory gating is reduced in healthy controls under stressful or fearful conditions [23,24], conditions that increase state anxiety levels. Interestingly, parent-reported difficulties in attention and anxiety in infants of 40 months old predicted sensory gating related suppression deficits to the repeating stimulus [25]. This means that anxiety-driven problems with inattention and sensory inhibition to distracting events can be observable from childhood.

In this study, we investigated the influence of preclinical anxiety in healthy participants measured with the State and Trait Anxiety Inventory [26] in pre-attentive processes governed by the auditory roving oddball paradigm, as shown with EEG. Trait anxiety is considered a stable personality characteristic, whereas state anxiety is more of a transitory response to a situation [27]. It has been argued that trait anxiety influences state anxiety levels, whereas state anxiety negatively associates with cognitive performance [27]. Here, we are interested about the effects of anxiety measures on change detection (MMN) and sensory gating when performing the roving oddball during EEG under emotionally neutral conditions. Based on previous neuroimaging research in anxiety patients and studies using the STAI in the healthy population, we expected anxiety to display hypervigilant responses to deviant stimuli and decreased suppression to the second repeating tone (S2) in a stimulus train in the roving oddball task.

## 2. Methods

### 2.1. Participants and Self-Report Measures

Data were collected from thirty-six healthy participants from 18 to 59 years of age (*M* = 32.06, *SD* = 12.40), of which 50/50 were of either gender. The sample size was determined accordingly with studies that examine the effects of anxiety at the oddball task [7,23,28], with N = 15, N = 36, and N = 23, respectively, and one other study that uses the same oddball version [5], with N = 40. Exclusion criteria consisted of: (i) lifetime history of mental disorder or substance use disorder, (ii) reported head injury, and (iii) intake of prescribed psychiatric medication. Participants provided written informed consent prior to their inclusion in the study, and the study was approved by the Psychology Department Ethics Committee of City, University of London (PSYETH (S_L) 16_17 06).

Self-reported measures of anxiety were collected using the STAI [26] questionnaire (Table 1). The State-Trait Anxiety Inventory (STAI) is a 40-item self-report devised by Spielberger et al. [26]. It is used to measure state and trait measures of anxiety in patients and in a healthy population, which result from its two forms, Y-1 and Y-2, respectively, each consisting of 20 items. The STAI has been previously shown to be a reliable psychometric scale with Cronbach’s alpha > 0.7 [29]. Responses for each item have a four-point scale: “not at all”, “somewhat”, “moderately so”, and “very much so”. The STAI was administered by a trained psychologist prior to the EEG recording.

### 2.2. Stimuli and Design

We recorded EEG activity using the same auditory roving oddball paradigm, as in previous studies [4,30]. This paradigm consists of stimulus trains of changing (roving) sinusoidal tones that range in frequency from 500 Hz to 800 Hz in random steps with integer multiples of 50 Hz. Within each stimulus train, all tones were of one frequency and were followed by a train of a different frequency. The first tone of a train was a deviant, which eventually became a standard after few repetitions. This meant that deviants and standards had the same physical properties, differing only in the number of times they had been presented. The number of times a tone of the same frequency was presented varied pseudo-randomly between one and eleven. The probability that the same tone was presented once or twice was 2.5%; for three and four times, the probability was 3.75%; and for five to eleven times, it was 12.5%. Each tone was presented through a speaker positioned on the left-hand side next to the computer monitor for 70 ms, with 5 ms rise and fall times and an interstimulus interval (ISI) of 500 ms. Repeated tones of a specific frequency were included within each stimulus train and were followed by a sequence of tones with different frequency. The first tone in a stimulus train was considered the deviant (about 185 trials) and standard after six or more repetitions (approximately 190 trials).

Concurrently with the oddball paradigm, participants performed a distracting visual task, a fixation cross changed color from black to grey and vice versa. The instruction given to participants was to allocate their attention to the color-changing cross presented in the middle of the screen and respond to it by pressing the “space” button of the keyboard standing in front of them with their right index finger. The use of the visual task was to keep participants active during EEG recording and eliminate alpha waves as much as possible. We were interested to examine processes of change detection (MMN) and sensory gating at the absence of active attentional allocation to the auditory roving oddball paradigm as per the literature [7,16]. Color change occurred in a pseudo-random ISI of 2 to 5 s and did not overlap with the tone frequency changes. The duration of the entire paradigm was 15 min.

### 2.3. EEG Acquisition and Processing

EEG was recorded with a 64-channel BrainVision BrainAmp series amplifier (Brain Products, Herrsching, Germany; 63 active electrodes in a ActiCAP 64Ch EEG cap) with a 1000 Hz sampling rate, filtered using a 300 HZ anti-aliasing filter. Data were recorded with reference to the FC_z_ electrode and the ground electrode at AF_z_. Electrooculographic (EOG) signal was recorded with an electrode placed below the left eye. All pre-processing steps were carried through in BrainVision Analyzer (Brain Products, Herrsching, Germany); during pre-processing, EEG data were down sampled to 250 Hz, and the high-pass was filtered at 0.5 Hz, which has been shown to improve independent component analysis decomposition [31]. We corrected for ocular movements with an automated independent component analysis (ICA) procedure using the mean sloped algorithm Gratton et al. [32], then re-referenced to TP_9_ and TP_10_ mastoid electrodes.

**Figure 1:**
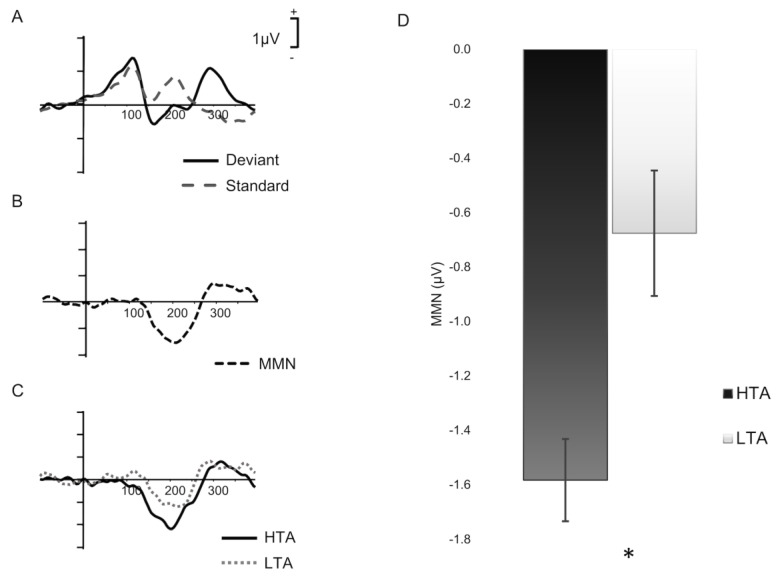
(**A**) Deviant (continuous black line) and standard (dashed grey line) stimuli ERP waveforms in the entire sample (N = 36). (**B**) MMN waveform (deviant minus standard) at Fz electrode site for all participants and (**C**) for HTA (continuous line) versus LTA (dotted line) groups. (**D**) Bar plot shows the MMN amplitude at Fz between HTA and LTA subjects following median split; * indicates significant differences; error bars represent standard error of the mean. Abbreviations: HTA: high-trait anxiety LTA: low-trait anxiety; μV: microvolt; MMN: mismatch negativity.

For the MMN, data were segmented for (i) the first new tone in a stimulus train (deviant) that followed a stimulus train with six or more repetitions of the previous tone and (ii) for the sixth repetition (standard) in a peristimulus window of 500 ms spanning from −100 ms pre to 400 ms. Deviant and standard segments were lowpass filtered (with infinite impulse response -IIR- filters) at 50 Hz, with a 12 dB/oct slope. Using automatic artifact rejection, we excluded segments with a slope of 50 μV/ms, min–max difference of 200 μV in a 200 ms interval, and low activity of 0.5 μV in a 100 ms interval. Before averaging, data were baseline corrected using the 100 ms interval preceding the stimulus. We considered the MMN as the difference wave of the standard stimulus from the deviant one at Fz electrode site in the 130–230 ms time window (Figure 1), in line with studies using the same roving oddball paradigm [4]. Approximately 190 standard and 185 deviant segments were used for averaging per participant.

For sensory gating, following the re-referencing step mentioned above, we segmented the EEG data into a peristimulus window of 1050 ms, from −100 ms to 950 ms post stimulus, containing the first (S1) and second (S2) tones of same frequency in a new stimulus train. We applied slightly different preprocessing steps from the MMN for sensory gating because we used peak detection similar to other studies of sensory gating [16]. Segments containing artifacts of −50 μV to 50 μV were automatically detected and rejected before being baseline corrected and averaged. An average of 200 artifact-free segments (min–max range = 110–240) containing S1 and S2 were used for averaging per participant. Finally, averaged segments were band-pass filtered from 10 Hz to 50 Hz (IIR) (12 dB/oct slope) to prevent aliasing [16]. We performed peak detection at the C_z_ electrode, at the most positive deflection occurring between 40–80 ms and 540–580 ms for S1 and S2, respectively. We then subtracted the highest preceding negativity between 30–60 ms and 530–560 ms from the positive peak at S1 and S2, respectively. We then calculated the difference (S1–S2) and ratio (S2/S1) between the two peaks. To exclude outliers, we used a maximum ratio of 2 [16]. We calculated the square root for the amplitudes of S1 and S2 to achieve a normal distribution in these variables for our analysis. There were three subjects in our sample without a positive S1 amplitude (no preceding negativity at 30–60 ms), which were excluded from the analysis; therefore, sensory gating analyses corresponded to a sample of thirty-three participants. Participants without an evident positive S2 peak were considered to have completely suppressed response to the repeating stimulus and were assigned the value of 0.01 μV [16].

### 2.4. Statistical Analysis

Statistical analysis of the mean amplitudes of the MMN and sensory gating were conducted in SPSS (SPSS 25, Armonk, NY, USA: IBM Corp). For the MMN, we first performed a paired-sample *t*-test for stimulus type (deviant/standard) to evaluate whether the roving oddball paradigm was successful in eliciting change detection in our experimental setup. We then performed partial correlations between state and trait anxiety with MMN, standard, and deviant stimulus amplitudes at the Fz electrode site while controlling for trait and state anxiety, respectively. Bayesian summary statistics were applied to the data to completement the correlation analysis. We further performed ANCOVA for the MMN amplitude at Fz with group (high-trait/low-trait anxiety OR high-state/low-state anxiety) as a fixed factor while controlling for the other anxiety subtype (state anxiety OR trait anxiety as a covariate, respectively). The high and low state/trait anxiety groups were created by median split of state (*mdn* = 32.5) and trait (*mdn* = 41) scores. As shown in Table 1, subjects with scores above the median were included in the “high-“ group, and those with scores below the median were in the “low-“ group, similar to previous neuroimaging studies [33].

For sensory gating, we performed partial correlations between state/trait anxiety measures and S1 and S2 amplitude, their difference (S1–S2), and ratio (S2/S1), controlling for trait/state anxiety measures, respectively. Following up significant correlations, we again used median split to evaluate group level differences with ANCOVA while covarying for either subtype of anxiety.

## 3. Results

### 3.1. Questionnaire Data

A total of thirty-six subjects (50% females, mean age = 32 years old) were recruited and completed the STAI questionnaire and EEG during the oddball paradigm. Mean scores on the STAI measures, as well as demographics, are reported in Table 1. State and trait anxiety were positively correlated with each other (*r* = 0.537, *p* < 0.001). Age and sex did not associate significantly with either of the STAI measures (*p* > 0.216).

### 3.2. MMN

A paired-sample *t*-test indicated a strong effect of stimulus (*t*_(33)_ = −6.954, *p* = 4.376 × 10^−8^, *Cohen’s d* = −1.159), indicating successful change detection between the deviant and standard stimuli in our experimental setup. Figure 1A shows the waveforms for deviant and standard stimuli, and 1B shows the MMN waveforms in the entire sample.

Trait anxiety did not correlate with standard (*p* = 0.556) or deviant (*p* = 0.196) amplitudes, covarying for state anxiety. MMN amplitude was significantly negatively correlated with trait anxiety, when controlling for state anxiety at the Fz site (*r*_p_ = −0.426, *p* = 0.011; two-tailed), demonstrating that higher trait anxiety levels show enhanced (more negative) MMN amplitudes (Figure 1C). We complemented the Pearson correlation with Bayesian summary statistics, providing moderate evidence for the alternative two-tailed hypothesis that the MMN amplitude correlated with trait anxiety (BF_10_ = 5.223)

To illustrate the differences in MMN wave as a function of trait anxiety, we split the group in high and low trait anxiety based on the median of the entire sample (*mdn* = 41). As the literature suggests, a cut off of 40 is generally considered to discriminate between probable clinical levels of anxiety [34]. We also validated these differences using ANCOVA, with trait anxiety as group factor, and state anxiety as a covariate. As Figure 1C shows, participants who scored higher in trait anxiety (>41) had an enhanced MMN wave, whereas participants with low trait anxiety had more attenuated amplitudes. This was also confirmed by the ANCOVA, which showed a significant main effect of trait anxiety factor in the MMN (*F*_(1,33)_ = 14.259, *p* = 6.323 × 10^−6^, *η_p_^2^* = 0.302) (Figure 1D). Thus, as is shown in Table 2, high-trait anxious individuals had significantly enhanced (negative) MMN amplitudes. MMN differences between high- and low-trait anxiety groups were driven by more negative amplitudes for deviant tones in high-trait anxiety. However, they were not significantly different to those of low-trait anxious individuals (*p* = 0.096) (Table 2).

Partial correlations for state anxiety while controlling for trait were not significant for neither standard (*p* = 0.366), deviant (*p* = 0.942), or MMN (*p* = 0.277) amplitudes.

### 3.3. Sensory Gating

**Figure 2:**
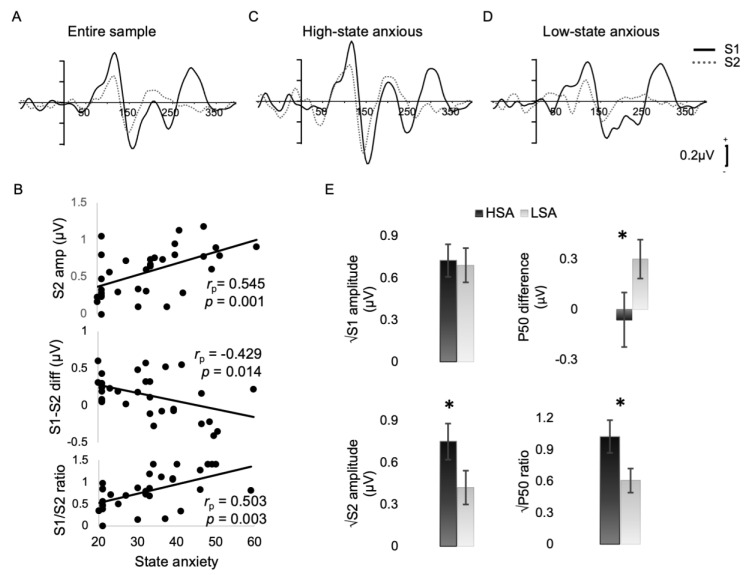
Grand average waveforms for the S1 (continuous line) and S2 (dotted line) stimuli at Cz superimposed onto each other for (**A**) the entire sample, (**B**) the subaverage of the high-state anxious, and (**C**) low-state anxious participants. (D) Scatterplots displaying the linear associations of the S2 amplitude, P50 difference, and P50 ratio with state anxiety on the right-hand side. Partial correlation coefficients and associated *p*-values show the strength of these correlations and the probability these were observed due to chance. (**E)** Bar plots for the sensory gating parameters (S1 and S2 amplitude, S1–S2 difference and S2/S1 ratio), showing the differences between HSA and LSA groups following median split; * indicates significant differences; error bars represent standard error of the mean. Abbreviations: HSA: high-state anxiety; LSA: low-state anxiety; ms: milliseconds; μV: microvolt; S1: stimulus S1; S2: stimulus S2.

Pre-attentive sensory gating was analyzed at the C_z_ electrode. The identical repeated stimulus (S2) was successfully gated in our participant sample as a whole, as revealed by paired-sample *t*-test that showed a significant reduction to S2 amplitude compared to S1 (*t*_(32)_ = 2.520, *p* = 0.017, *Cohen’s d* = 0.439) in the entire sample, therefore suggesting suppression of the repeated stimulus in our experimental setup (Figure 2A).

Significant partial correlations (two-tailed) were detected with state anxiety and S2 amplitude (*r_p_* = 0.545, *p* = 0.001), S1–S2 difference (*r_p_* = −0.429, *p* = 0.014), and S2/S1 ratio (*r_p_* = 0.503, *p* = 0.003) while controlling for trait anxiety (Figure 2B). Summary Bayesian statistics showed strong evidence for the alternative hypothesis against the null, which was that state anxiety correlated significantly with S2 amplitude and S2/S1 ratio (BF10 = 37.326, BF10 = 15.380) and moderately with S1–S2 difference (BF = 4.235).

For illustration purposes, we followed up significant correlations of the sensory gating ERPs with state anxiety, and we median split participants into groups of high- and low-state anxiety. This was performed to visually explore the groups’ differences and to also validate these differences statistically using ANCOVA while controlling for trait anxiety. Sub-average ERP waveforms for S1 and S2 are shown in Figure 2C for high-state anxious participants and in Figure 2D for low-state anxious individuals. Low-state anxious participants had significantly lower S2 amplitude (*F*_(1,30)_ = 9.102, *p* = 0.005, *η_p_^2^* = 0.233) compared to high-state anxious participants. Additionally, suppression to the S2 stimulus was significantly different between the groups as seen by both the S1–S2 difference (*F*_(1,30)_ = 10.642, *p* = 0.003, *η_p_^2^* = 0.262) and S2/S1 ratio (*F*_(1,30)_ = 13.117, *p* = 0.001, *η_p_^2^* = 0.304) indicating lack of suppression to the S2 stimulus in the high-state anxiety group at the Cz electrode site (Table 3; Figure 2E). Accordingly, early auditory response to the S2 stimulus was not filtered in the high-state anxious individuals, whereas low-state anxious participants successfully gated the repeated S2 tone by suppressing the response to it.

None of the sensory gating parameters correlated significantly with trait anxiety (*p* > 0.060) while controlling for state anxiety.

## 4. Discussion

In this study, we explored the impact of self-reported anxiety, utilizing the STAI, on ERP activity during the auditory roving oddball paradigm. Our aim was to assess state and trait anxiety effects on auditory change detection and information filtering. Our results showed that higher trait anxiety increased MMN waves, whereas state anxiety reduced sensory gating.

High-trait anxious participants showed more negative responses to deviant tones compared to low-trait anxious participants. Under the predictive coding hypothesis, the failure of top-down connections to suppress prediction error causes strengthening of the bottom-up ones, resulting in strong error detection and MMN [4]. Hence, MMN is mediated by a complex interplay of top-down and bottom-up connection dynamics [4], with frontal feedback signals mediating attentional reorienting and temporal feedforward connections regulating sensory memory encoding [3,35]. This bidirectional modulation is affected in high-trait anxiety, resulting in a hypervigilant neurophysiological response against the oddball stimulus at the pre-attentive level.

The lack of diminished top-down modulation and bottom-up connectivity enhancement in anxiety [36], together with impaired prefrontal activity in high-trait anxious individuals [37], potentially drive increased sensitivity and hyperarousal to deviant stimuli that are perceived as more salient. These mechanisms could then compromise attentional capture that affect attentional shifting and control, as bottom-up processes are prioritized over top-down ones to drive attention at the deviant stimulus [38]; in our experiment, such impairment could be expressed as a heightened pre-attentive MMN response, as is also evidenced by trait [6,7], state [28], or anticipatory anxiety [8] or in individuals with anxiety disorders, such as PTSD [9,10,11] and panic disorder [12].

On the other hand, sensory gating has been shown to correlate with attentional control [39] manifested as increasing S1 and decreasing S2 amplitudes when healthy participants are asked to allocate their attention to the stimuli [40]. In addition, inhibitory processes govern the suppression of the repeated stimulus [41]. Indeed, supporting evidence reveals associations of sensory gating and goal-directed attention, as it significantly correlates with latent inhibition and sustained attention processes [42]. Early sensory gating could reflect a combination of top-down and bottom-up processes, allowing one to identify stimulus irrelevance and orient selective attention towards the relevant ones [42]. Such inhibition was argued to be related with cognitive mechanisms (rather than sensory/motor ones), as its neural substrates are located in forebrain regions, (i.e., prefrontal, cingulate, and parietal areas), revealed by electrophysiological recordings in epileptic patients [43]. Therefore, diminished suppression to the repeated stimulus can be a cause of sensory overload due to insufficient filtering of irrelevant stimuli, as it increases attention to distraction [44]. Furthermore, by demonstrating an effect of state anxiety on sensory gating, we extend previous studies reporting that state anxiety only affected bottom-up attention processes [45] since sensory gating is a process involving an interplay of top-down and bottom-up dynamics [46].

State anxiety is regarded as a transient state influenced by trait anxiety and environmental effects on mood [37]. Our results point out that individual differences in state anxiety are marked by increases in S2 amplitudes (similar to clinical population), which suggest failure to gate out the redundant S2 stimuli. Individuals with schizophrenia exhibit impairments in P50 suppression that can either be expressed as failure to gate in or gate out the auditory stimuli, by exhibiting smaller S1 amplitudes [47,48] or larger S2 [49], respectively; specifically, S2 amplitude-driven differences in a P50 ratio indicates an impairment driven by lack of inhibition to the repeating stimulus [22]. P50 suppression abnormalities like those of schizophrenic patients are found in individuals with anxiety disorders such as panic disorder [18,19], PTSD [20,50,51], and obsessive compulsive disorder [52]. S2 amplitude-driven sensory gating differences support the notion that the sensory gating impairment explains deficits in information filtering rather than registration. This effect has been shown to anti-correlate with benzodiazepine use in panic disorder [19]. Accordingly, anxiety-related sensory gating impairment could potentially be reversed with administration of anxiolytic drugs, supporting the idea that gating deficits could be generated by states of mental distress.

Similar to our results, sensory gating reduction has been observed under physical stress-inducing conditions, an effect driven by enhanced S2 amplitude [23]. However, our study provides evidence that impaired inhibitory control can arise under neutral conditions and without explicitly inducing stressful conditions. Thus, we showed that inhibitory processes of attentional control can be compromised under acute worrying states (heightened state anxiety), and we expand current knowledge regarding the same effects under threatening conditions [53] or in relation to increased trait anxiety [54] only.

To conclude, there are two main findings in this study. First, the MMN response was enhanced (more negative) in high-trait anxious versus low-trait anxious participants at the Fz electrode site. Second, suppression to the S2 stimulus is compromised in high-state anxious participants, which is shown by significant differences in both the peak difference and ratio between high- and low-state anxiety groups at the Cz electrode site. The sensory gating deficit in high-state anxious participants is driven by the S2 amplitude that is significantly increased in this group of participants who fail to gait out the repeated tone. Trait anxiety drives hypervigilant attention shifting to deviant stimuli (MMN) as they become more salient, whereas state anxiety is responsible for diminished inhibitory control and failure to suppress redundant information. We have also showed that heightened levels of state and trait anxiety in healthy individuals bear similarities with anxiety disorders with regard to MMN and sensory gating. Under a neuroimaging perspective, state and trait anxiety share commonalities and differences; even though the two measures are highly correlated with each other, they are often found to each have distinct effects in cognitive neurophysiological processes measured with the EEG [55,56,57]. Such is the case in our study, both state and trait anxiety tap into attentional processing; however, the former has an effect on inhibiting or gating redundant information, which could be a more transient (state) effect, and the latter associates with hypervigilant detection of salient features, a change in a potentially more stable (trait) nature.

## Figures and Tables

**Table 1:** Demographics and questionnaire characteristics for all participants and anxiety subgroups.

	All Participants	High-Trait Anxious	Low-Trait Anxious	High-State Anxious	Low-State Anxious	Statistics
N	36	17	19	19	17	
Age (SD)	32.06 (12.40)	29.59 (11.66)	36.43 (12.88)	32.95 (14.12)	31.06 (10.48)	HTA vs. LTA: nsHSA vs. LSA: ns
Sex (F:M)	18:18	10:7	8:11	12:7	6:11	HTA vs. LTA: nsHSA vs. LSA: ns
STAI						
State anxiety	33.06 (10.53)	38.53 (10.32)	28.32 (8.40)	41.21 (7.02)	23.94 (4.44)	
Trait anxiety	42.08 (11.35)	51.29 (7.42)	33.74 (6.93)	47.11 (7.95)	36.35 (12.08)	

F: Female; HSA: High-State Anxious; HTA: High-Trait Anxious; LSA: Low-State Anxious; LTA: Low-Trait Anxious; M: Male; N: Sample size; ns: Not Significant; SD: Standard Deviation; STAI: State-Trait Anxiety Inventory.

**Table 2:** Trait anxiety group characteristics for MMN.

	Trait Anxiety Mean (SD)		Statistics	
	High (N = 17)	Low (N = 19)	*p*	*η_p_* ^2^
Age	29.59 (11.66)	34.26 (12.93)		
Sex (F:M)	(10:7)	(11:8)		
Mismatch negativity (Fz)				
Deviant–Standard difference (μV)	−1.58 (0.62)	−0.68 (1.01)	<0.001	0.299
Deviant (μV)	−0.87 (1.21)	−0.06 (1.30)	0.096	0.081
Standard (μV)	0.77 (1.03)	0.62 (1.27)	0.447	0.017

F: female; Fz: Fz electrode; M: male; μV: microvolt; MMN: mismatch negativity; N: sample size; *η*_*p*_^2^: partial eta-squared; SD: standard deviation.

**Table 3:** State anxiety group characteristics for sensory gating.

	State Anxiety Mean (SD)		Statistics	
	High (N = 16)	Low (N = 17)	*p*	*η_p_^2^*
Age	34.25 (14.93)	31.06 (10.48)		
Sex (F:M)	9:7	6:11		
Sensory gating (Cz)				
S1 amplitude (μV)	0.73 (0.21)	0.69 (0.26)	0.885	0.001
S2 amplitude (μV)	0.75 (0.27)	0.42 (0.25)	0.005	0.233
S1–S2 difference (μV)	−0.024 (0.31)	0.27 (0.17)	0.003	0.262
S2/S1 ratio	1.03 (0.39)	0.61 (0.23)	0.001	0.304

Cz: Cz electrode; F: female; M: male; μV: microvolt; N: sample size; *η*_*p*_^2^: partial eta-squared; SD: standard deviation.

## Data Availability

The data presented in this study are available on request from the corresponding author. The data are not publicly available due to ethical issues.
